# Impact of Hyperbaric Oxygen on More Advanced Wagner Grades 3 and 4 Diabetic Foot Ulcers: Matching Therapy to Specific Wound Conditions

**DOI:** 10.1089/wound.2018.0855

**Published:** 2018-12-20

**Authors:** William J. Ennis, Enoch T. Huang, Hanna Gordon

**Affiliations:** ^1^ Catherine and Francis Burzik Professor Wound Healing and Tissue Repair, University of Illinois at Chicago, Chicago, Illinois.; ^2^ Hyperbaric Medicine and Wound Care, Legacy Emanuel Medical Center, Portland, Oregon.; ^3^ Research and Informatics, Healogics, Inc., Jacksonville, Florida.

**Keywords:** diabetic foot ulcer, hyperbaric oxygen therapy, advanced wound therapy, Wagner grade 3 or 4, adjunctive wound therapy

## Abstract

**Objective:** The goal of this research was to identify a population of diabetic foot ulcer patients who demonstrate a significant response to hyperbaric oxygen therapy (HBOT) using a large sample size to provide guidance for clinicians when treating these complicated patients.

**Approach:** The effect of HBOT on diabetic foot ulcers, Wagner grades 3 and 4, was evaluated using a retrospective observational real-world data set. The study reported on the overall healing rate, (74.2%) at the population level, for >2 million wounds.

**Results:** When a subgroup of patients of only foot ulcers with a Wagner grade 3 or 4 were considered, the healing rate was only 56.04%. The use of HBOT, without filtering for the number of treatments received, improved the healing rate to 60.01% overall. Healing rates for this same subgroup, however, were improved to 75.24% for patients who completed the prescribed number of hyperbaric treatments.

**Innovation:** This observational study discusses the importance of reporting at the population level, specific wound etiology level, a risk-stratified level, and to then overlay the effect of treatment adherence on those outcomes to provide clinicians with a comprehensive understanding of when to prescribe an advanced modality such as hyperbaric oxygen.

**Conclusion:** The authors provide healing outcomes data from several prior HBOT studies as well as other advanced modalities that have been used in diabetic foot ulcer care for comparison and context.

**Figure:**
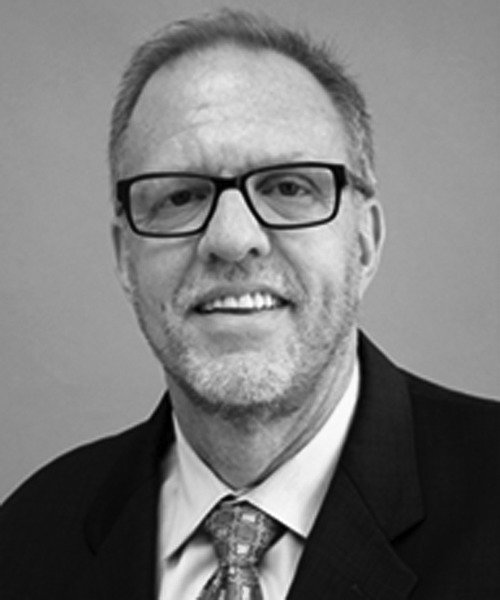
William J. Ennis, DO, MBA, MMM, CPE

## Introduction

There has been much debate in the literature surrounding the overall benefits of hyperbaric oxygen therapy (HBOT) in wound care.^1,2^ Many of the initial studies that resulted in positive outcomes, payment policies, and physician adoption were performed in hospital settings ensuring compliance and thereby, not surprisingly, the results did not translate to an outpatient clinic reality. Studies also reported various primary outcome objectives making comparisons difficult while confusing clinicians when confronted with an individual case.^3–5^ Even when outcomes were assessed for wounds of a single etiology, there was little effort to risk stratify the patients either for their overall clinical condition or for the complexity of their specific wound. Although population level wound-healing rates have been reported, stratified outcomes data are needed based on specific wound etiology to provide insight when making individual treatment decisions. A modified intent-to-treat (mITT) healing rate for >1 million wounds was recently reported at 74.6%.^6^ This article did not, however, describe the granular healing rates for individual wound etiologies. When diabetic wound-healing rates are reported, they can be an aggregate of diabetic wounds of the leg or diabetic foot ulcers of various Wagner grades. In many of these studies, when HBOT is given, the total number of treatments completed is rarely considered, making the impact of the therapy difficult to assess.

The primary focus of this study is to report on the mITT outcomes of HBOT on diabetic ulcers limited to the foot and specifically only the more complex Wagner grades 3 and 4 using the world's largest wound care database. The goal of this research was to identify a population of diabetic foot ulcer patients that demonstrate a significant response to HBOT using a large sample size to provide guidance for clinicians when faced with treating these complicated patients. In addition, this study expands the previously reported mITT wound-healing outcomes to provide continued updates on the outcomes of patients who were still in treatment, and therefore excluded, at the conclusion of the last study period. The wounds in the study are an extension of the previously reported data and, therefore, any concerns about the final outcomes of those patients still in treatment at the end of the last study period should be answered by a similar healing rate and the large sample size in the present trial. Finally, this report adds granularity into specific wound etiology healing outcomes at a population level. Specifically, we studied diabetic foot ulcers that were Wagner grade 3 or 4.

## Clinical Problem Addressed

There has been much debate in the literature surrounding the overall benefits of HBOT in wound care.^1,2^ Many of the initial studies that resulted in positive outcomes, payment policies, and physician adoption were performed in hospital settings ensuring compliance and thereby, not surprisingly, the results did not translate to an outpatient clinic reality. Studies also reported various primary outcome objectives, making comparisons difficult while confusing clinicians when confronted with an individual case.^3–5^ This observational study discusses the importance of reporting at the population level, specific wound etiology level, a risk-stratified level, and to then overlay the effect of treatment adherence on those outcomes to provide clinicians with a comprehensive understanding of when to prescribe an advanced modality such as hyperbaric oxygen.

## Materials and Methods

The initial phase of this study was to review and update the retrospective data on wounds, HBOT, and the final clinical disposition from 682 outpatient wound care centers nationwide between January 1, 2014, and April 28, 2018. The time frame for data inclusion was determined by the availability of aggregate data at the time of analysis. The data were obtained from a proprietary clinical database and collected using a specialized wound data capture system that tracks wound-related treatments and patient outcomes. Nurses and physicians document visits at the point of care. A subset of centers document using paper-based forms, which were then entered into a central system at the end of each work day. Other centers document visits on a fully electronic medical record basis. The data used for the study were compiled into a deidentified research database table distinct from the enterprise data warehouse, before the beginning of the analysis. All patient identifiers were removed from the research file. Deidentified data were extracted using SQL software and analyzed using Stata 14.1. The study was exempt from IRB review by Quorum Review IRB (QR no. 33110).

All centers in the sample were managed by a wound care management company and staffed by a provider panel that consisted of a combination of contract physicians in private practice and a subset of employed providers who practiced wound care full-time. All providers whether contracted or affiliated undergo a formal standardized course in wound healing that includes the management of diabetic foot ulcers before providing care at a wound center. All programs are hospital based and have program directors, managers, nurses, and access to hyperbaric oxygen, and needed specialty consultants. All providers who order and utilize HBOT have completed at a minimum a 40-h course approved by either the Undersea Hyperbaric Medical Society or the American College of Hyperbaric Medicine. All diabetic patients in either HBOT or standard of care only received care based on evidence-based clinical practice guidelines that are used at all centers. All patients, whether offered HBOT or not, undergo glycemic control, debridement as needed, off-loading, appropriate wound care dressings, assessment and revascularization if needed, control of bioburden and, overall management of their comorbid medical conditions. All patients regardless of wound etiology undergo a medical surveillance review process every 4 weeks throughout the course of their wound care treatment to identify patients who are not healing along an anticipated trajectory. Specifically, patients who are being considered for HBOT undergo pretreatment reviews of the medical record to ensure that standard of care was met and that patients did not show meaningful signs of improvement before starting their treatment.

The next phase of the analysis was to create an analytic subsample of diabetic wounds. All Wagner grade 3 or 4 diabetic ulcers that were located on the foot or toe were assessed. The decision to only utilize wounds located on the foot and toe was to focus the outcomes on purely diabetic foot ulcers and not the broader category of diabetic wounds of the lower extremity (DWLE), also an approved HBOT indication. As previously stated, most of the initial HBOT trials were limited to wound locations below the ankle, making more direct comparisons from this study to the existing published literature possible. The sample was further limited to cases in which a single wound was noted to ensure the ability to accurately identify the ulcer for which HBOT was prescribed, including those in active treatment at the time of study closure. The final population size included 25,562 diabetic foot ulcers. The study reports retrospective observational data on healing and amputation outcomes using a mITT framework for outcomes measurement. Additional information regarding the mITT model for outcomes in wound healing has been reported elsewhere, but briefly represents the percentage of all nonactive nonconsultation wounds, with greater than 7 days between first and last assessment, that were healed. In addition, population level healing results were collected and reported by wound etiology. This allows clinicians to put healing rates in context with other types of wound care cases. Cases were then compared by the variable of either receiving or not receiving HBOT. Physicians in the centers contributing data in this study are trained to provide the best standard of care for a minimum of 30 days to assess for a positive healing response. Those who fail to improve are considered potential candidates for several advanced modalities, including HBOT. There would likely be more heterogeneity in the DWLE population as diabetic patients might have ischemic wounds, venous ulcers, or traumatic wounds located somewhere on the lower extremity, which might be coded as having a diabetic etiology. The software used by providers in this study has fields that allow for the documentation of both the primary wound etiology and concomitant medical conditions that could contribute to the patients overall healing capacity. To focus on the potential impact of HBOT on diabetic foot ulcers, the study restricted the location of the wound to below the ankle.

## Results

During the study time frame, a total of 2,651,878 wounds were evaluated (Table 1). The population level mITT healing rate was 74.2%, which is consistent with the previously reported 74.6% based on 1,006,690 wounds at the time of that publication. There was variability in the specific wound mITT healing rates from 55.3% to 80.6%. Not surprisingly, arterial wounds demonstrated the lowest healing rates and venous leg ulcers healed at the highest level. The overall healing rate for all wounds classified as diabetic was 70.9% (328,158/462,888) at the population level. At this level of stratification, there does not seem to be a major difference in the overall healing rate for the aggregated overall population of wounds and the rate of healing specifically diabetic wounds using the mITT method previously described. Only patients with a single wound of Wagner grades 3 and 4 located on the foot or toe were included for additional study. The healing and amputation rates for the full sample of Wagner grades 3 and 4 diabetic foot ulcers are reported in Table 2. Once the mITT exclusions are applied, the sample is reduced to 19,057 ulcers with a 56.04% healing rate and a 4.09% amputation rate. This rate is comparable with the mITT population level healing rate for arterial ulcers. By comparison, the mITT healing rate for all wound etiologies previously published by Ennis *et al.* was 74.6%.^6^ The lower healing rate for Wagner grades 3 and 4 is an indication of the difficulty in healing these patients who often have confounding medical comorbid conditions and emphasizes the importance of risk stratification when reporting outcomes. The mITT subpopulation represents 75% of the total population of diabetic foot ulcer patients with the largest group excluded being those still in treatment at the end of the study time frame, which accounted for 18.1% of the total.

**Table 1.** Wound healing rates by etiology and aggregate

*mITT 2014–2018*	*Arterial*	*Diabetic*	*Pressure*	*Venous*	*All Wound Types*
Total no. of healed wounds	34,745	328,158	190,832	296,219	1,408,871
Total no. of wounds	89,469	605,102	447,064	475,203	2,651,878
% Healed at population level	38.83	54.23	42.69	62.34	53.13
Exclude—no. of active treatments at study conclusion	4,516	8,544	32,406	8,331	87,098
% of total	5.05	1.41	7.25	1.75	3.28
No. of remaining wounds	84,953	596,558	414,658	466,872	2,564,780
% Healed at level	40.90	55.01	46.02	63.45	54.93
Exclude—no. of without wound documented	24	320	349	402	6,227
% of total	0.03	0.05	0.08	0.08	0.23
No. of remaining wounds	84,929	596,238	414,309	466,470	2,558,553
% Healed at level	40.91	55.04	46.06	63.50	55.07
Exclude—no. of consult and with days first to last assessment ≤7 days	22,049	133,350	116,073	99,078	658,735
% of total	24.64	22.04	25.96	20.85	24.84
Final—no. of remaining wounds	62,880	462,888	298,236	367,392	1,899,818
% Healed at level mITT	55.26	70.89	63.99	80.63	74.16
% Amputation at level mITT	2.99	2.42	0.5	0.11	0.94

mITT, modified intent-to-treat.

**Table 2.** Modified intent-to-treat healing rate and amputation rate: diabetic single wound Wagner grade 3/4 on foot or toe

	*All Single Wound 3/4*
Total no. of wounds	25,562
% Healed at population level	43.65
Exclude—no. of active at study conclusion	1,877
% of total	7.34
No. of remaining wounds	23,685
% Healed at level	46.39
Exclude—no. of without wound documented	0
% of total	0
No. of remaining wounds	23,685
% Healed at level	46.39
Exclude—no. of consult and with days first to last assessment ≤7 days	4,624
% of total	18.10
Final—no. of remaining wounds	19,057
% Healed at level mITT	56.04
% Amputated at level mITT	4.09

**Figure 1.**
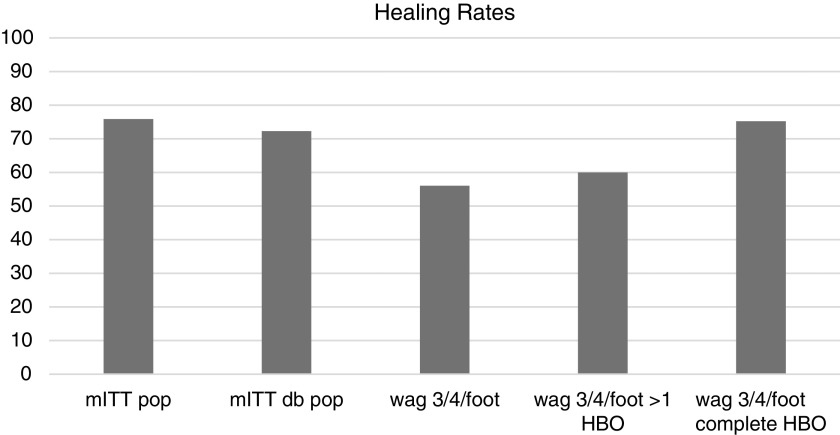
Healing Rates. mITT pop: modified intent-to-treat population level. mITT db pop: modified intent-to-treat diabetic population. wag 3/4/foot: Wagner Grade 3 or 4 on foot. wag 3/4/foot>1 HBO: Wagner Grade 3 or 4 on foot incomplete HBOT. wag 3/4/foot complete HBO: Wagner Grade 3 or 4 on foot completed HBOT treatment course. HBOT, hyperbaric oxygen therapy; mITT, modified intent-to-treat.

Table 3 reports further granular outcomes for patients who received at least one HBOT treatment (6,616) compared with patients who did not receive any HBOT (18,946). After the same mITT exclusions are applied, the patients who received HBOT demonstrated a slightly higher mITT healing rate (60.01%) than patients who did not receive HBOT (54.33%), which results in a 9.47% delta. The mITT amputation rates were consistent between the two groups with a 4.16% amputation rate in the HBOT sample and a 4.06% amputation rate in the non-HBOT group. This improvement in healing rate, however, does not take into account the actual amount of HBOT received or whether the patients completed their entire overall clinical course of care. Table 4 analyzes the patients' treatment based on whether the patients completed their entire clinical treatment protocol divided into those who did or did not receive HBOT. More patients who received HBOT went on to complete their entire wound care treatment protocol. The wound treatment protocol refers to the entire course of therapy that patients receive during their care at the wound center. For example, a patient might receive HBOT and still undergo several more weeks of advanced wound care before reaching a final discharge disposition. Patients who commit to such an intensive therapy such as HBOT, requiring every day treatments for up to 8 weeks, are likely to also be more committed to completing the entire course of therapy. This correlation could also, however, prove to be a confounder and represent a surrogate marker for healthier patients or those with more adherence to their treatment plans. This is a descriptive retrospective study using big data and although correlation may not equal causality, we further analyzed the demographics and wound characteristics for the two groups (HBO and non-HBO) to further identify the potential impact for the therapeutic intervention. Of the 5,742 patients who received HBOT, only 2,597 completed their hyperbaric treatment (45.2%); however, of those patients who did receive a full HBOT course, 75.24% versus 47.44% were healed for a delta of 36.9% (Table 5). These data provide insight into why there are differences in the literature describing efficacy outcomes and how the therapy potentially might lose effectiveness in the outpatient clinic setting when patients fail to adhere to the full treatment regimen. Patients who complete their HBOT received 89% of the prescribed treatments, whereas those marked as incomplete only received 57% of their prescribed treatments (Table 6). The reasons for incomplete HBOT are further detailed in Table 7. These options are preloaded drop-down choices built into the software. The most common reason for an incomplete treatment course was indicated as “Patient Choice” followed by “Wound Progress Plateaued.” Patients who did not complete the ordered treatment course on average only completed 57% (standard deviation [SD] 34) of the mean 38 treatments (SD 12) ordered and discontinued treatment an average of 40 days after the first HBOT treatment (Table 7). Overall, patients who were marked as having chosen to discontinue treatment by their own request completed the lowest percentage of treatments (40%; SD 31), whereas patients marked as “Wound Progress Plateaued” completed the highest percentage of ordered treatments with an average of 88% (SD 21) of the mean 40 treatments ordered (SD 13). A hypothesis to explain this phenomenon is that a provider might continue to treat a patient with the goal of establishing a positive healing trajectory and would, in that case, want to ensure a full course of HBOT was administered before deeming the treatment ineffective. Further information from patients who choose to quit is needed for future studies. Another group that did not complete the course of HBOT are those in which the wound healed during the treatment course. For obvious reasons, this group had no clinical reason to complete their course of therapy. When treatment adherence is not included (data separated by any HBOT vs. no HBOT), the previously noted 60.01% healing rate was observed. Stated another way, patients demonstrated a 23% improvement delta when HBOT is delivered as ordered and the overall treatment plan is adhered to. Of course, this assumes that the patient was medically stabilized, revascularization was performed if indicated and clinically possible, infection was controlled, offloading provided, and the wound received debridement when indicated before, during, and after HBOT was delivered. It is, therefore, imperative that future research includes patient adherence information to fully appreciate the therapeutic benefit achieved with HBOT. These data can also help support patient engagement opportunities, not unlike those employed in pharmaceutical industry to assist providers in achieving the best possible outcomes when using HBOT or any other modality in which total dosing is important. These tables clearly identify two variables critical to considerations of the effectiveness of HBOT in real-world samples: first patients need to complete their overall care in the wound center as HBOT is only adjunctive to good care, and second, when HBOT is ordered, it is critical to complete the course of therapy. Figure 1 identifies mITT healing rates for various subgroups and provides background for topics covered in the discussion section that follows.

**Table 3.** Modified intent-to-treat healing rate and amputation rate

	*HBO*	*No HBO*
Total no. of wounds	6,616	18,946
% Healed at population level	53.30	40.29
Exclude—no. of active at study conclusion	490	1,387
% of total	7.41	7.32
No. of remaining wounds	6,126	17,559
% Healed at level	56.58	42.83
Exclude—no. of without wound documented	0	0
% of total	0	0
No. of remaining wounds	6,126	17,559
% Healed at level	56.58	42.83
Exclude—no. of consult and with days first to last assessment ≤7 days	382	4,242
% of total	5.77	22.39
Final—no. of remaining wounds	5,742	13,315
% Healed at level mITT	60.01	54.33
% Amputated at level mITT	4.16	4.06
9.47% delta		

Hyperbaric oxygen vs. nonhyperbaric oxygen diabetic Wagner grade 3/4 single wound located on the foot or toe.

HBO, hyperbaric oxygen.

**Table 4.** Admission marked as “completed treatment” by hyperbaric oxygen therapy status

	*HBO*	*No HBO*	*Total (%)*	N
Admission—completed treatment	64.56	56.8	59.14	11,270
Admission—treatment incomplete	35.44	43.2	40.86	7,787
Total	100	100	100	19,057

**Table 5.** Modified intent-to-treat by hyperbaric oxygen therapy course completion hyperbaric oxygen therapy group only

	*Healed (%)*	*Not Healed (%)*	*Total (%)*	N
Complete HBOT treatment course	75.24	24.76	45.23	2,597
Incomplete treatment course	47.44	52.56	54.77	3,145
Total	60.01	39.99	100	5,742

HBOT, hyperbaric oxygen therapy.

**Table 6.** Summary statistics for hyperbaric oxygen therapy sample by hyperbaric oxygen therapy course completion

	*Treatments*	*Treatments*	*Percentage Complete*	*Days First to Last HBOT*
Complete HBOT treatment course				
Mean	40.14	36.44	0.89	61.91
Std.	13.35	15.63	0.22	30.35
Median	40	35	1	57
Incomplete treatment course				
Mean	37.78	22.62	0.57	40.66
Std.	12.00	17.51	0.34	32.70
Median	40	20	1	36
Total				
Mean	38.85	28.87	0.71	50.09
Std.	12.68	18.05	0.33	33.39
Median	40	30	1	48

**Table 7.** Summary statistics for hyperbaric oxygen therapy sample by reason for incomplete treatment course, hyperbaric oxygen therapy incomplete only

	*Treatments Ordered*	*Treatments Complete*	*Percentage Complete*	*Days First to Last HBOT*
Death				
Mean	36.47	14.72	0.40	25.53
SD	12.90	14.26	0.32	28.60
Median	30.00	8.50	0.27	19.00
Early resolution				
Mean	37.28	21.77	0.57	38.90
SD	10.19	12.73	0.26	24.72
Median	40.00	20.00	0.57	36.00
Financial				
Mean	36.44	20.79	0.55	40.34
SD	10.55	13.35	0.29	29.43
Median	39.00	20.00	0.60	38.00
Medical complication				
Mean	36.24	16.10	0.43	29.40
SD	10.24	13.57	0.29	28.04
Median	30.00	13.00	0.40	23.00
Patient choice				
Mean	36.01	14.74	0.40	29.77
SD	10.06	13.79	0.31	31.00
Median	30.00	11.00	0.30	22.00
Wound progress plateaued				
Mean	40.95	36.99	0.88	62.87
SD	13.11	15.50	0.21	29.96
Median	40.00	38.00	1.00	58.00
Total				
Mean	37.65	22.51	0.57	40.66
SD	11.32	17.01	0.34	32.70
Median	40	20	1	36
N	3145	3145	3145	3145

SD, standard deviation.

## Discussion

This retrospective study suggests that HBOT can be effective for hard-to-heal Wagner grades 3 and 4 diabetic foot ulcers and demonstrates the complexities of studying the therapy using observational real-world data. Specifically, the results underscore the importance of treatment adherence when analyzing the effectiveness of HBOT. Furthermore, using the mITT framework to report healing outcomes allows for both transparency of results and the ability to compare programs, individual centers, and ultimately providers. Although the population level healing rate provides an overall picture of the effectiveness of wound care centers in general, we also need to analyze results on more granular levels. Venous ulcer healing rates, for example, are frequently reported without segregation into various clinical, etiology, anatomy, and pathophysiology (CEAP) classifications, making it difficult to project an individual patients potential for healing.^7^ Arterial ulcer healing rates rarely describe the level and extent of peripheral arterial disease when reporting healing rates. In addition, the methods of establishing revascularization are often not a variable that is considered in the final analysis. Given the fact that HBOT is approved for DWLE, a highly heterogeneous group, separating wounds by anatomic location and Wagner grade may provide different results. Variations in diabetic foot ulcer healing rates have been reported based on hospital designation, that is, community versus tertiary academic center, further complicating how results are interpreted.^8^ In that study, the same clinical team provided care using the same protocols at two very different hospital settings. The first, a small community 200-bed hospital and the second, a 700-bed level one trauma tertiary setting. The noted difference in healing rates at these two centers (73.7% vs. 59.5%) achieved by the same clinicians sheds light on patient referral patterns and risk stratification.

There have been several randomized studies that have noted improvements in healing rates of diabetic foot ulcers. A few highly cited articles are described herein. Löndahl *et al.* published a randomized single-center double-blinded placebo-controlled trial in 2010.^9^ The study was conducted in an outpatient setting using a multiplace chamber. Patients received either oxygen or air at 2.5 atmospheres of pressure. The investigators did, however, include Wagner grade 2 ulcers (24% of the cases) in this study, making comparisons with the present study more difficult. More patients healed in the intent-to-treat group (*p* < 0.03); however, this effect was improved in a per-protocol subgroup, when >35 treatments were received (*p* < 0.009). This fact supports the findings of this study in that total treatments received matters to the overall outcome. The study was designed for a 1-year time frame and significance was achieved at 9 months. Kessler *et al.* randomized 28 patients with diabetic foot ulcers who were admitted to an inpatient hospital unit to receive HBOT or standard of care. The patients were given two HBOT treatments per day, 5 days a week for 2 weeks.^10^ All patients had normal vascular examinations before enrollment. There was a significant reduction in wound area at the end of the 2 weeks in the HBOT patients, but upon discharge the significant improvement was lost as both groups improved similarly. The HBOT impact in the first 2 weeks was significant given that both groups were receiving intense inpatient management and the only differential treatment was the use of HBOT. Questions raised by this trial design include why was the healing trajectory benefits of early HBOT lost once the HBOT was discontinued. Duzgan *et al.* demonstrated a 66% healing rate compared with 0% in an inpatient setting treating infected diabetic foot ulcers.^11^ The protocol included two treatments per day followed by one treatment a day on a basis for 20–30 days. Many subsequent reviewers were troubled by the control group having no patients healed. The inpatient setting likely had an impact on diet, offloading, medication adherence, and glucose control, all of which are more difficult to manage in the outpatient setting. In addition, the patients were not in the hospital for >1 month, so it is not unreasonable that the healing rate for this site of care would not be comparable with an outpatient trial, for example. Abidia *et al.* studied nonreconstructable vascular patients with a diabetic foot ulcer and found statistically improved healing at 6 weeks and 1 year.^12^ The protocol was daily HBOT at 2.4 air pressure absolute, 5 days a week for 30 treatments. Interestingly in this study even though the ulcer dimensions in the control group became smaller, they did not go on to healing at the 1 -year mark. This concept has been discussed by the FDA as a reason that wound-healing studies need to include total closure as one of the outcomes to ensure that early rapid healing does not actually negatively impact the ultimate outcome of healing.^13^ This is also why studies looking at alternative surrogate markers for healing need to ensure that complete healing is in fact predicted by the earlier time frame-based surrogate.^14^ Kalani *et al.* reported on 38 patients with nonreconstructable vascular disease and diabetic foot ulcers for a 3-year period.^15^ HBOT-treated patients reported a 76% healing rate compared with 48% in the control arm. Patients received between 40 and 60 treatments. In all of these studies, there was a positive trend for HBOT when healing outcomes are measured. The problem with all of the studies, however, is the variation in HBOT treatment frequency, total number of HBOT treatments, small sample size, various sites of care that impacted adherence, and variations in major comorbid conditions such as infection and vascular status. The published outcomes of other advanced modalities, for example, ultrasound therapy, have also been challenged due to various protocols, dosing regimens, and the use of various wound etiologies without risk adjustment being used in a single trial.^16^

Not all studies have found a positive correlation with the use of HBOT and the healing of diabetic foot ulcers however. As with the literature purporting a positive impact of HBOT, the publications that found no effect have limitations as well. Margolis *et al.* published a retrospective review of a large database using propensity scoring.^17^ There was a median of 29 treatments delivered in the HBOT arm but no description of healing rates correlating with the number of treatments actually received. This study on a cohort of 6,259 patients failed to demonstrate an improvement in healing for nonischemic diabetic foot ulcers. An article published in 2016 that used a double-blinded sham protocol for diabetic foot ulcer treatment with HBOT found no statistical reduction in the recommendation for amputation.^18^ Surprisingly, these patients did not actually receive amputations, they were simply evaluated by a single surgeon through photographs and a decision for amputation was created at that point. There was much disagreement with this study as documented by published letters to the editor requesting further clarifications.^19^

A Cochrane review also failed to support HBOT but noted the positive trends in wound healing in the short term but not the long term and recommended additional, higher quality studies to be performed in the future.^20^

The importance in selecting an appropriate delta is critical when doing a power calculation to determine the total number of patients needed for a study. Given the paucity of consistent findings in the HBOT literature, this poses a challenge to researchers conducting power calculations for studies of HBOT. As a result, studies that are often conducted using underpowered designs or clinical criteria for inclusion may be extended to patients who would not typically benefit from the therapy to meet the needed sample size. Recent literature describes the risks of using random methods to assign delta values and the bias introduced by doing so. A recent trial conducted at several centers in the Netherlands evaluated diabetic foot ulcers with a study powered to an anticipated delta of 12% improvement of limb salvage. When enrollment failed to meet targets, the delta was increased to a 25% limb salvage benefit and a 29% improvement in healing.^21^ By arbitrarily doubling the expected delta, the findings of the study are at substantial risk of bias and unlikely to detect a significant effect of the therapy. Although the authors clearly articulate this limitation, its nuance may be overlooked by clinical audiences. In addition to the underpowered study design, patients were combined and crossed over due to patient preference, leaving only 39 patients who completed HBOT. Despite these shortcomings, the trends were all in favor of HBOT. It is of interest to note that in this study, only 65% of patients undergoing HBOT were able to complete their course of treatment. A per-protocol analysis did show that the patients who completed HBOT did have statistically significantly less amputations and higher amputation-free survival. This is consistent with the findings in this report.

Providers have other advanced treatment options when caring for diabetic foot ulcer patients. There have been few treatments, however, that have undergone rigorous clinical trials at the randomized controlled trial (RCT) level. Guidelines have been proposed by several professional societies for treatment of diabetic foot ulcers.^22,23^ Most guidelines recommend performing standard of care for at least 1 month before considering any of the advanced modalities. The percentage area reduction in 1 month has repeatedly been found to be a useful surrogate for predicting those patients who will likely go on to heal compared with those in which advanced treatments should be considered.^24–26^ The providers practicing in the centers whose data comprise this study are all trained to provide at least 1 month of standard of care, and to monitor wound-healing trajectories before using advanced therapies. All coverage and reimbursement criteria include these standards as well. A well-documented method of off-loading for diabetic foot ulcer patients is the use of total contact casting. Despite having strong evidence to support its effectiveness, total contact casting is not frequently used in many wound care centers.^27,28^ Once a patient has been identified as being hard to heal, and having failed standard of care, the clinician has several options that have been studied for the treatment of diabetic foot ulcers. Becaplermin, a recombinant platelet-derived growth factor product, was the first drug available for diabetic foot ulcers and underwent several prospective randomized placebo-controlled trials and a meta-analysis.^29–31^ In the meta-analysis, 50% of ulcers versus 36% with placebo gel healed at 20 weeks (28% delta). The patients in these trials, however, were well perfused and were clinically assessed as Wagner grade 2, making it difficult to compare with the findings from this study. Dermagraft, a human cellular-based product, produced a higher percentage (30%) of healed ulcers compared with controls (18%) in a single-blinded RCT that enrolled 314 patients (40% delta). Again in this trial, well-perfused Wagner grade 2 wounds were evaluated.^32^ Apligraf, a human cellular bilayered construct, demonstrated a 56% versus 38% healing against controls at 12 weeks of therapy (32% delta).^33^ Negative pressure wound therapy has also been studied with respect to diabetic foot ulcer healing.^34^ The studies evaluating negative pressure have looked at surgical diabetic wounds and chronic wounds, whereas the advanced modalities have focused primarily on more superficial well-perfused noninfected Wagner grade 2 ulcers. Although achieving significance in efficacy trials, many of these methods have not performed as well in effectiveness evaluations due to the heterogeneity of the patients seen in a real-world clinical setting.

Needless to say, this study is a descriptive observational study and as such has limitations that must be recognized. It does, however, double the number of wounds available for healing outcomes, and by doubling our prior work makes this the largest study of its kind. The clinical procedures, policies, and protocols at the clinical sites have now consistently achieved similar repeatable healing outcomes at an aggregate rolled up population level, which implies adherence to the agreed-upon clinical practice guidelines developed by the company. This is a retrospective analysis and as such carries all the standard potential for bias that observational studies are known to be vulnerable to. However, the purpose of the study was to leverage a large database and standard outcomes reporting framework to identify the directionality and magnitude of possible treatment effects. Using the mITT method of outcomes reporting provides a consistent comparable measure by which to compare and analyze results and to more realistically calculate treatment effects that are achievable in an outpatient setting.

In Table 8, the overall wound-healing rate at the population level is reported as 75.9% and this drops to 72.3% when the data set is further modified to include the condition of diabetes. When the more advanced Wagner grades 3 and 4 diabetic ulcers located on the foot are used as filters, the healing rate drops to 56.04%. The use of any HBOT brings that value up to 60.01%, but when only completed HBOT cases are evaluated, the healing rate is 75.24%. Although big data analysis identifies correlations, it does not imply causation, so one could argue that those patients who complete their HBOT are healthier, have less comorbid conditions that would make completing their HBOT more likely, or any number of other hypotheses. A randomized study by Faglia *et al.* found a significant reduction in amputations when using HBOT, again in a controlled inpatient environment where treatment adherence was extremely high, providing further support for both the treatment and the need to complete the course of therapy.^35^ For the purposes of this descriptive analysis, we stratified ulcers using the Wagner scale. However, there are currently a number of broader scoring systems that warrant consideration for future studies as well as other clinical characteristics that should be measured in subsequent analyses.^36,37^

**Table 8.** Summary characteristics

	*HBOT*			*No HBOT*		
	*Mean/%*	*Standard Deviation*	*Median*	*Mean/%*	*Standard Deviation*	*Median*
Wound area at first assessment	7.66	14.79	2.25	6.51	12.68	1.95
Wound duration at first assessment (days)	83.28	103.66	38	80.62	107.21	31
Patient age	61.16	12.36	61	62.59	13.37	62
Female (%)	30.44			33.31		

A further consideration should be to balance the cost of care relative to the likelihood of healing and the likelihood that the patient will complete treatment. If we make the case that Wagner grade 3 or 4 wounds on the foot that do not respond to standard of care can be healed at the overall rate for all wounds, we need to know how much we need to spend to achieve this clinical parity. What about recidivism? Overall mortality? The results of this big data analysis identify a potential set of patients for whom HBOT might provide a substantial improvement in wound healing. Other available advanced modalities have been proven to achieve significant improvements over controls for less severe cases and might offer a good option for those cases. Future studies should more completely explore questions related to patient adherence and possible incremental benefits of HBOT. Patients need to be engaged and adherent in order for this treatment approach to work. We are now looking at gaining more granularity into the vascular status of these patients, their social determinants of health, cost of care, and their recurrence rates over time to continue to provide the most value-based wound care possible for this complex group of patients. Ultimately, we need to use big data to help create value-based algorithms of care along a spectrum of various clinical complexities.

## Innovation

This retrospective study has clinical relevance because it suggests HBOT can be effective for hard-to-heal Wagner grades 3 and 4 diabetic foot ulcers and demonstrates the complexities of studying the therapy using observational real-world data. Specifically, the results underscore the importance of treatment adherence when analyzing the effectiveness of HBOT. Furthermore, using the mITT framework to report healing outcomes allows for both transparency of results and the ability to compare programs, individual centers, and ultimately providers.

Key Findings

- HBOT can be effective for hard-to-heal Wagner grades 3 and 4 diabetic foot ulcers.
- Results underscore the importance of treatment adherence when analyzing the effectiveness of HBOT.
- Using an mITT framework to report healing outcomes allows for both transparency of results and the ability to compare programs, individual centers, and ultimately providers.

## About the Authors

**William J. Ennis, DO, MBA, MMM, FACOS,** is Catherine and Francis Burzik Professor of Wound Healing and Tissue Repair and Chief of the Section of Wound Healing Tissue Repair at the University of Illinois at Chicago. Dr. Ennis is a founding board member and President of the American College of Wound Healing and Tissue Repair, a 501c3 nonprofit educational organization that has a mission of bringing wound care to the level of a formal medical specialty. He also serves as Chief Medical Officer of Healogics, Inc., a wound care company based in Jacksonville, FL. **Enoch T. Huang, MD, MPH&TM, FUHM, FACEP,** received his MD and MPH&TM from Tulane University in New Orleans. He completed a fellowship in Undersea and Hyperbaric Medicine at the University of Pennsylvania. He is boarded in both Emergency Medicine and Undersea and Hyperbaric Medicine. He is the past president of the Undersea and Hyperbaric Medical Society. **Hanna Gordon, PhD*****,*** received her PhD from Florida State University in 2014. She currently serves as the Executive Director of Research and Informatics at Healogics, Inc., a wound care company based in Jacksonville, FL.

## Acronyms and Abbreviations

DWLE: diabetic wounds of the lower extremity
HBOT: hyperbaric oxygen therapy
mITT: modified intent-to-treat
RCT: randomized controlled trial

## References

[1] Dauwe PB, Pulikkottil BJ, Lavery L, Stuzin JM, Rohrich RJ Does hyperbaric oxygen therapy work in facilitating acute wound healing: a systematic review. Plast Reconstr Surg 2014;133:208e–215e 10.1097/01.prs.0000436849.79161.a4 24469192

[2] Chuck AW, Hailey D, Jacobs P, Perry DC Cost-effectiveness and budget impact of adjunctive hyperbaric oxygen therapy for diabetic foot ulcers. Int J Technol Assess Health Care 2008;24:178–183 18400121 10.1017/S0266462308080252

[3] Doctor N, Pandya S, Supe A Hyperbaric oxygen therapy in diabetic foot. J Postgrad Med 1992;38:112–114, 111. 1303408

[4] Chen CY, Wu RW, Hsu MC, Hsieh CJ, Chou MC Adjunctive hyperbaric oxygen therapy for healing of chronic diabetic foot ulcers: a randomized controlled trial. J Wound Ostomy Continence Nurs 2017;44:536–545 28968346 10.1097/WON.0000000000000374

[5] Kaur S, Pawar M, Banerjee N, Garg R Evaluation of the efficacy of hyperbaric oxygen therapy in the management of chronic nonhealing ulcer and role of periwound transcutaneous oximetry as a predictor of wound healing response: A randomized prospective controlled trial. J Anaesthesiol Clin Pharmacol 2012;28:70–75 22345950 10.4103/0970-9185.92444 PMC3275977

[6] Ennis WJ, Hoffman RA, Gurtner GC, Kirsner RS, Gordon HM Wound healing outcomes: Using big data and a modified intent-to-treat method as a metric for reporting healing rates. Wound Repair Regen 2017;25:665–672 28846162 10.1111/wrr.12575

[7] Padberg FT Jr CEAP classification for chronic venous disease. Dis Mon 2005;51:176–182 15900270 10.1016/j.disamonth.2005.03.013

[8] Ennis WJ, Lee C, Vargas M, Meneses P Wound outcomes from a single practice at a Sub-acute wound care unit and 2 hospital based, outpatient settings. Wounds 2004;16:165–172

[9] Löndahl M, Katzman P, Nilsson A, Hammarlund C Hyperbaric oxygen therapy facilitates healing of chronic foot ulcers in patients with diabetes. Diabetes Care 2010;33:998–1003 20427683 10.2337/dc09-1754 PMC2858204

[10] Kessler L, Bilbault P, Ortéga F, et al. Hyperbaric oxygenation accelerates the healing rate of nonischemic chronic diabetic foot ulcers: a prospective randomized study. Diabetes Care 2003;26:2378–2382 12882865 10.2337/diacare.26.8.2378

[11] Duzgun AP, Satır HZ, Ozozan O, et al. Effect of hyperbaric oxygen therapy on healing of diabetic foot ulcers. J Foot Ankle Surg 2008;47:515–519 19239860 10.1053/j.jfas.2008.08.002

[12] Abidia A, Laden G, Kuhan G, et al. The role of hyperbaric oxygen therapy in ischaemic diabetic lower extremity ulcers: a double-blind randomised-controlled trial. Eur J Vasc Endovasc Surg 2003;25:513–518 12787692 10.1053/ejvs.2002.1911

[13] FDA Wound Healing Clinical Focus Group. Guidance for industry: chronic cutaneous ulcer and burn wounds—developing products for treatment. Wound Repair Regen 2001;9:258–268 11679134 10.1046/j.1524-475x.2001.00258.x

[14] Driver VR, Gould LJ, Dotson P, et al. Identification and content validation of wound therapy clinical endpoints relevant to clinical practice and patient values for FDA approval. Part 1. Survey of the wound care community. Wound Repair Regen 2017;25:454–465 28370922 10.1111/wrr.12533

[15] Kalani M, Jörneskog G, Naderi N, Lind F, Brismar K Hyperbaric oxygen (HBO) therapy in treatment of diabetic foot ulcers. Long-term follow-up. J Diabetes Complications 2002;16:153–158 12039398 10.1016/s1056-8727(01)00182-9

[16] Ennis WJ, Lee C, Gellada K, Corbiere TF, Koh TJ Advanced technologies to improve wound healing: electrical stimulation, vibration therapy, and ultrasound—what is the evidence? Plast Reconstr Surg 2016;138(3S):pp.94S–104S 27556780 10.1097/PRS.0000000000002680

[17] Margolis DJ, Gupta J, Hoffstad O, et al. Lack of effectiveness of hyperbaric oxygen therapy for the treatment of diabetic foot ulcer and the prevention of amputation: a cohort study. Diabetes Care 2013;36:1961–1966 23423696 10.2337/dc12-2160 PMC3687310

[18] Fedorko L, Bowen JM, Jones W, et al. Hyperbaric oxygen therapy does not reduce indications for amputation in patients with diabetes with nonhealing ulcers of the lower limb: a prospective, double-blind, randomized controlled clinical trial. Diabetes Care 2016;39:392–399 26740639 10.2337/dc15-2001

[19] Huang E Comment on Santema et al. Hyperbaric oxygen therapy in the treatment of ischemic lower-extremity ulcers in patients with diabetes: results of the DAMO2CLES multicenter randomized clinical trial. Diabetes Care 2018;41:112–119. Diabetes care 2018;41:e61 29074815 10.2337/dc17-0654

[20] Kranke P, Bennett MH, James MS, Schnabel A, Debus SE, Weibel S Hyperbaric oxygen therapy for chronic wounds. Cochrane Database Syst Rev 2015;CD004123 26106870 10.1002/14651858.CD004123.pub4 PMC7055586

[21] Santema KT, Stoekenbroek RM, Koelemay MJ, et al. Hyperbaric oxygen therapy in the treatment of ischemic lower-extremity ulcers in patients with diabetes: results of the DAMO2CLES multicenter randomized clinical trial. Diabetes Care 2018;41:112–119 29074815 10.2337/dc17-0654

[22] Hingorani A, LaMuraglia GM, Henke P, et al. The management of diabetic foot: a clinical practice guideline by the Society for Vascular Surgery in collaboration with the American Podiatric Medical Association and the Society for Vascular Medicine. J Vasc Surg 2016;63(2 Suppl):3S–21S 26804367 10.1016/j.jvs.2015.10.003

[23] Huang ET, Mansouri J, Murad MH, et al. A clinical practice guideline for the use of hyperbaric oxygen therapy in the treatment of diabetic foot ulcers. Undersea Hyperb Med 2015;;42:205–247 26152105

[24] Sheehan P, Jones P, Caselli A, Giurini JM, Veves A Percent change in wound area of diabetic foot ulcers over a 4-week period is a robust predictor of complete healing in a 12-week prospective trial. Diabetes Care 2003;26:1879–1882 12766127 10.2337/diacare.26.6.1879

[25] Lavery LA, Barnes SA, Keith MS, Seaman JW, Armstrong DG Prediction of healing for postoperative diabetic foot wounds based on early wound area progression. Diabetes Care 2008;31:26–29 17934156 10.2337/dc07-1300

[26] Snyder RJ, Cardinal M, Dauphinée DM, Stavosky J A post-hoc analysis of reduction in diabetic foot ulcer size at 4 weeks as a predictor of healing by 12 weeks. Ostomy Wound Manage 2010;56:44–50 20368673

[27] Armstrong DG, Lavery LA, Wu S, Boulton AJ Evaluation of removable and irremovable cast walkers in the healing of diabetic foot wounds: a randomized controlled trial. Diabetes Care 2005;28:551–554 15735186 10.2337/diacare.28.3.551

[28] Armstrong DG, Short B, Espensen EH, Abu-Rumman PL, Nixon BP, Boulton AJ Technique for fabrication of an “instant total-contact cast” for treatment of neuropathic diabetic foot ulcers. J Am Podiatr Med Assoc 2002;92:405–408 12122129 10.7547/87507315-92-7-405

[29] Steed D.L Clinical evaluation of recombinant human platelet-derived growth factor for the treatment of lower extremity diabetic ulcers. Diabetic Ulcer Study Group. J Vasc Surg 1995;21:71–78; discussion 79–81. 10.1016/s0741-5214(95)70245-8 7823364

[30] Wieman TJ, Smiell JM, Su Y Efficacy and safety of a topical gel formulation of recombinant human platelet-derived growth factor-BB (becaplermin) in patients with chronic neuropathic diabetic ulcers. A phase III randomized placebo-controlled double-blind study. Diabetes Care 1998;21:822–827 9589248 10.2337/diacare.21.5.822

[31] Smiell JM, Wieman TJ, Steed DL, Perry BH, Sampson AR, Schwab BH Efficacy and safety of becaplermin (recombinant human platelet-derived growth factor-BB) in patients with nonhealing, lower extremity diabetic ulcers: a combined analysis of four randomized studies. Wound Repair Regen 1999;7:335–346 10564562 10.1046/j.1524-475x.1999.00335.x

[32] Marston WA, Hanft J, Norwood P, Pollak R The efficacy and safety of Dermagraft in improving the healing of chronic diabetic foot ulcers: results of a prospective randomized trial. Diabetes Care 2003;26:1701–1705 12766097 10.2337/diacare.26.6.1701

[33] Veves A, Falanga V, Armstrong DG, Sabolinski ML Graftskin, a human skin equivalent, is effective in the management of noninfected neuropathic diabetic foot ulcers: a prospective randomized multicenter clinical trial. Diabetes Care 2001;24:290–295.34. 11213881 10.2337/diacare.24.2.290

[34] Blume PA, Walters J, Payne W, Ayala J, Lantis J Comparison of negative pressure wound therapy using vacuum-assisted closure with advanced moist wound therapy in the treatment of diabetic foot ulcers: a multicenter randomized controlled trial. Diabetes Care 2008;31:631–636 18162494 10.2337/dc07-2196

[35] Faglia E, Favales F, Aldeghi A, et al. Adjunctive systemic hyperbaric oxygen therapy in treatment of severe prevalently ischemic diabetic foot ulcer: a randomized study. Diabetes care 1996; 19:1338–1343 8941460 10.2337/diacare.19.12.1338

[36] Mills Sr JL, Conte MS, Armstrong DG, et al. The Society for Vascular Surgery Lower Extremity Threatened Limb Classification System: risk stratification based on wound, ischemia, and foot infection (WIfI). J Vasc Surg 2013;59:220–234 e1–e2 24126108 10.1016/j.jvs.2013.08.003

[37] Lavery LA, Armstrong DG, Harkless LB Classification of diabetic foot wounds. J Foot Ankle Surg 1996;35:528–531 8986890 10.1016/s1067-2516(96)80125-6

